# The Buffalo Medical Journal

**Published:** 1845-07

**Authors:** 


*The Buffalo Medical Journal.*—This is the title of a medical journal, the first number of which was issued in the city of Buffalo, in June. It is edited by our late colleague, Austin Flint, M. D., under whose management it is certain of success, if that success be at all dependent upon the industry, talent, or acquirements of its editor. It contains 24 well printed octavo pages, with a neat cover, and is to be issued monthly, at the rate of $1,00 a year, in advance. The first two numbers, which are before us, contain a fair proportion of excellent original communications, and a variety of well selected matter. Among the former, we are pleased to notice the commencement of an interesting series of "Notes of a European Tour," by F. A. Hamilton, M. D., Prof. of Surgery in Geneva Medical College. We welcome with pleasure this accession to our exchange list, and wish the new Journal and its editor all the success which merit deserves.—Ed.

